# Clinical aspects of *Plasmodium falciparum* infection. Case presentation

**DOI:** 10.1186/1471-2334-13-S1-P60

**Published:** 2013-12-16

**Authors:** Bianca Voinescu, Mihaela Lupse

**Affiliations:** 1 Clinical Hospital of Infectious Diseases, Cluj-Napoca, Romania; 2 Department of Infectious Diseases, Iuliu Hațieganu University of Medicine and Pharmacy, Cluj-Napoca, Romania

## Background

The variability of clinical manifestation of *Plasmodium falciparum* (PF) infection remains a challenge in sub-Saharian countries, where clinical exam is the first diagnostic line.

The main objective of the presentations is to show the multitude of clinical aspects of PF infection.

I selected some cases from an indeterminate lot of patients who have been consulted and treated in Centre de Santé Buraniro, Province Kayanza, Burundi, Africa, between November 2012 – August 2013.

I choose for presentation: severe anemia among children, articular manifestation among teenagers, cerebral manifestation among elders, malnutrition and severe complication of malaria.

## Case report

1) Severe anemia in a 4 year-old child developed in less than 12 hours (in the first day of hospitalization capillary hemoglobin (Hb) was 13 g/dL; in the second day of hospitalization capillary Hb decreased to 5.4 g/dL).

2) Unexpected cerebral complication (profound coma) developed after 48 hours of hospitalization in an 82 year-old man who presented only asthenia-like clinical manifestation of malaria.

3) Several cases among teenagers with important arthralgia-like as the only clinical manifestation and with severe and fast evolution to death.

4) A 25 year-old woman with severe protein caloric malnutrition and unspecific clinical manifestation of malaria who developed pulmonary edema in less than 48 hours.

## Conclusion

The clinical outlook doesn't always represent a criterion for early diagnostic and for future complication of plasmodium infection.

Malaria remains an important problem in the world starting with the accuracy of clinical diagnosis, the necessity of laboratory confirmation, prevention and treatment of complications, social implications of morbidity and mortality.

